# Development and assessment of *CootVR*, a virtual reality computer program for model building

**DOI:** 10.1107/S2059798320013625

**Published:** 2021-01-01

**Authors:** Hamish Todd, Paul Emsley

**Affiliations:** a Huawei Research and Development, Cambridge, United Kingdom; b MRC Laboratory of Molecular Biology, Cambridge, United Kingdom

**Keywords:** computational crystallography, bioinformatics, visualization, model building, virtual reality

## Abstract

Biological macromolecules have complex three-dimensional shapes that are experimentally examined using X-ray crystallography and electron cryo-microscopy. Interpreting the data that these methods yield involves building 3D atomic models. With almost every data set, some portion of the time put into creating these models must be spent manually modifying the model in order to make it consistent with the data; this is difficult and time-consuming, in part because the data are ‘blurry’ in three dimensions. This paper describes the design and assessment of *CootVR* (available at http://hamishtodd1.github.io/cvr), a prototype computer program for performing this task in virtual reality, allowing structural biologists to build molecular models into cryo-EM and crystallographic data using their hands. *CootVR* was timed against *Coot* for a very specific model-building task, and was found to give an order-of-magnitude speedup for this task. A from-scratch model build using *CootVR* was also attempted; from this experience it is concluded that currently *CootVR* does not give a speedup over *Coot* overall.

## Abstract 2

Virtual reality-specific tools for model building are possible, and can provide an order-of-magnitude speedup over mouse-and-keyboard tools in certain situations.

## 1. Introduction   

**Figure 1:**
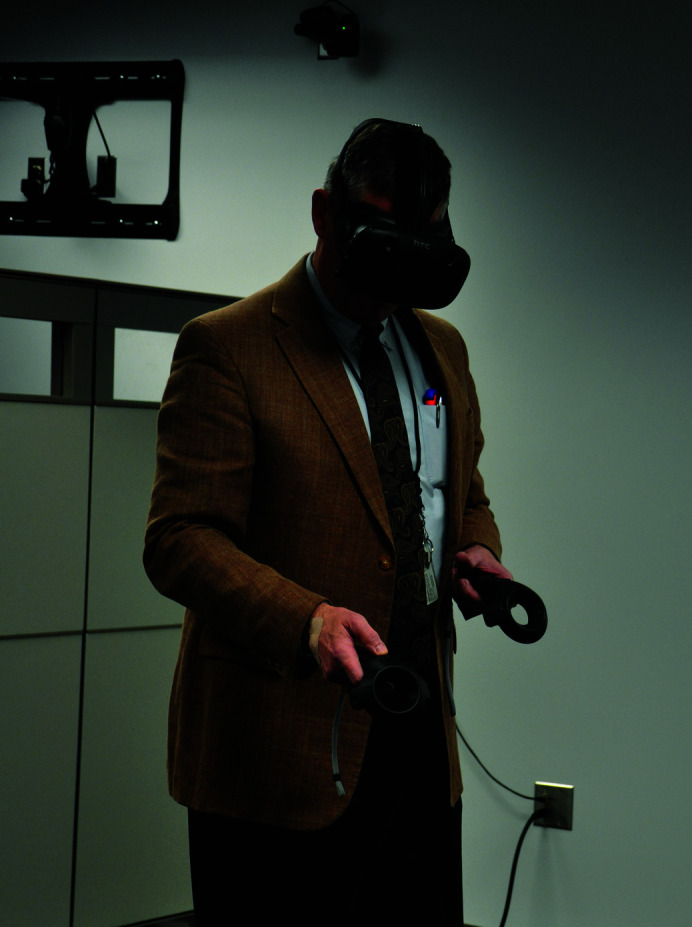
The HTC Vive in use, showing the headset and hand controllers. Behind the user a wall-mounted sensor can be seen, which some headsets still use (Teaching and Learning with Technology from USA, CC BY 2.0, via Wikimedia Commons)

Recently, consumer-priced hardware has become available that allows hand-tracked virtual reality (VR): devices that users can attach to their body and perceive complex geometry in 3D and manipulate this geometry using their hands (see Fig. 1 ▸).

**Figure 2:**
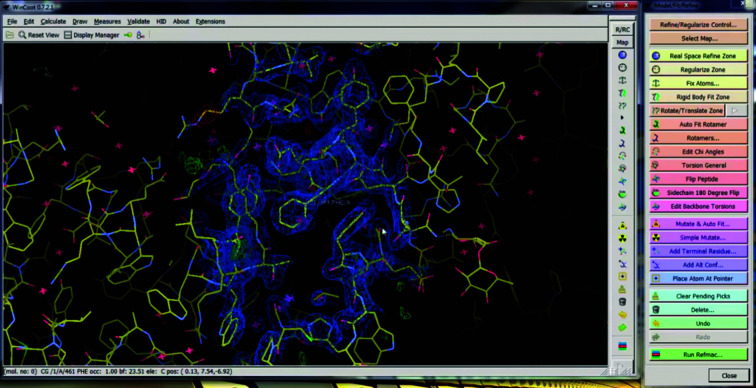
*Coot*, a program for manual model refinement. It has ordinary icons, windows and menus and a mouse-and-keyboard interface, together with a 3D visualization window.

Molecular model building has been an important task in structural biology since its inception, with the most famous example being Watson and Crick’s construction of the double-helix model (Watson & Crick, 1953 ▸). All biomolecular models still involve some amount of manual model building, because the data yielded by crystallography and cryo-EM are often at least somewhat noisy. *Coot* (Emsley *et al.*, 2010 ▸; Fig. 2 ▸) is the most widely used program for doing this. *Coot* is highly optimized for model building, which is necessary since operators will often use it for several hours at a time. It provides intuitive visualizations, many different tools and options, and useful keyboard shortcuts, to make model building as fast as possible.

**Figure 3:**
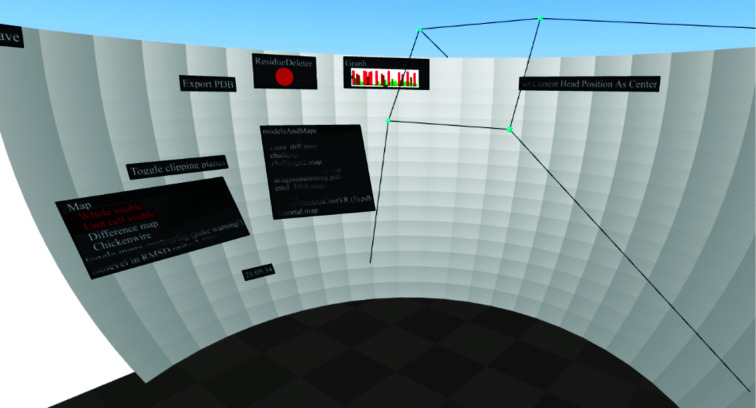
This figure depicts the user’s surroundings and is taken from an unusual point of view: ordinarily, the user’s head is at the center, at the point at the top of the truncated pyramid shape. The ‘shell’ shape is what we refer to as the panel.

The VR program *CootVR* (Fig. 3 ▸) was created for the purposes of finding out whether, and how, model building can be sped up using VR. A video of *CootVR* demonstrating several of its features is provided as supporting information and can also be viewed at https://www.youtube.com/watch?v=TdyYOWKDpGc. Its code is available at https://github.com/hamishtodd1/hamishtodd1.github.io/tree/master/cvr and can be used under the MIT license.

At the beginning of the project, the reasoning was that model building could be sped up by VR for the following three reasons.

(i) With hand controllers, the 3D positions of large groups of atoms could be specified simultaneously with a high amount of precision.
(ii) VR offers improved sense of the 3D shape of a molecule.
(iii) Improved ‘screen space’ for showing information on screen and navigating the large number of tools that might be needed.

Other minor advantages of VR were discussed, such as making it easier to teach model building and making it so that less wrist pain would be induced by the activity (the ‘*Coot* screen shake’ is a famous way for model builders to improve their 3D impression of a part of a model that they are looking at, although some complain of it inducing wrist pain). However, it was decided that the design, implementation and testing of *CootVR* would focus on the three properties described above.

Below, several ideas are described that we found to work very nicely in the context of a (specifically) VR program for structural biology: a new ‘selective visibility’ system, a simple but noteworthy molecule-navigating system, a ‘rigid mover’ tool and a ‘protein painter’ tool. We would claim that each of these developments represents an improvement on *Coot*. With the protein painter tool, this is indeed borne out by a performance test that we conducted that compared it with an equivalent approach in *Coot*.

However, it must be admitted that *CootVR* does not have many of the most commonly used functions that are needed in *Coot*, and the tools that it does offer do not fill in the gaps sufficiently, as was found out during a from-scratch model build using *CootVR* described below. Therefore, we do not recommend *CootVR* as a replacement for *Coot*. We do not recommend it as a supplement in most cases either, because current VR technology requires a significant amount of setup and maintenance that overwhelms all time savings. With this said, it is worth bearing in mind that augmented-reality headsets, which are expected to become common within the next decade (Oculus Connect 5|Keynote Day 01; https://www.youtube.com/watch?v=o7OpS7pZ5ok), will completely deal with the problem of maintenance and setup, and so we do believe that it is worth continuing to develop this technology.

*CootVR* is, to our knowledge, the only VR program to be specifically created for model building, although other studies (Goddard *et al.*, 2018 ▸) have discussed the possibility of the application. It is however far from the only VR program for working with biomolecules: other pieces of software have been created for use in figure-making (Goddard *et al.*, 2018 ▸), drug design (Norrby *et al.*, 2015 ▸), molecular docking (BioBlox; http://bioblox.org/) and education and presentation (Nanome; https://nanome.ai/), although none of them have experienced widespread adoption. Model building provides a very interesting example of a domain to which to apply VR, because in biomolecular model building 3D visualization has direct relevance to decisions that users are making up to dozens of times per minute. Additionally, users are constantly making changes to the structure that they are looking at. Therefore, significant time has been invested in optimizing these actions. It is therefore hoped that some of the designs will be applicable in different domains, and perhaps even beyond structural biology.

## 2. General interface   

Since it was designed for prolonged, professional usage, the layout of *CootVR* makes the presumption that the user is seated and looking forwards and slightly downwards (in contrast to the majority of VR programs, which involve walking around and looking in arbitrary directions).

Fig. 3 ▸ shows the ‘panel’. There are several different sorts of objects that can be placed on the panel.

(i) Non-interactive information; for example, what time of day it is.
(ii) Real-time-updating graphics; for example, a graph of the Ramachandran values of the amino acid nearest the user’s hand.
(iii) Interactive buttons, such as the ‘export pdb’ button.
(iiv) Tables, such as tables of binary options or lists of files in a directory that the user can ‘click on’ in order to load.
(v) ‘Tools’ that are to be ‘picked up’, such as the rigid mover and protein painter described below.
(vi) A fast way to navigate the model, such as the sequence view.

In *Coot* (but not *CootVR*), all of these functions would have their own window or button on the interface, following the ‘windows, icons, menus, pointers’ design pattern. The panel is very similar to this, with the only real change being the curvature and the increased amount of space (which is an advantage).

**Figure 4:**
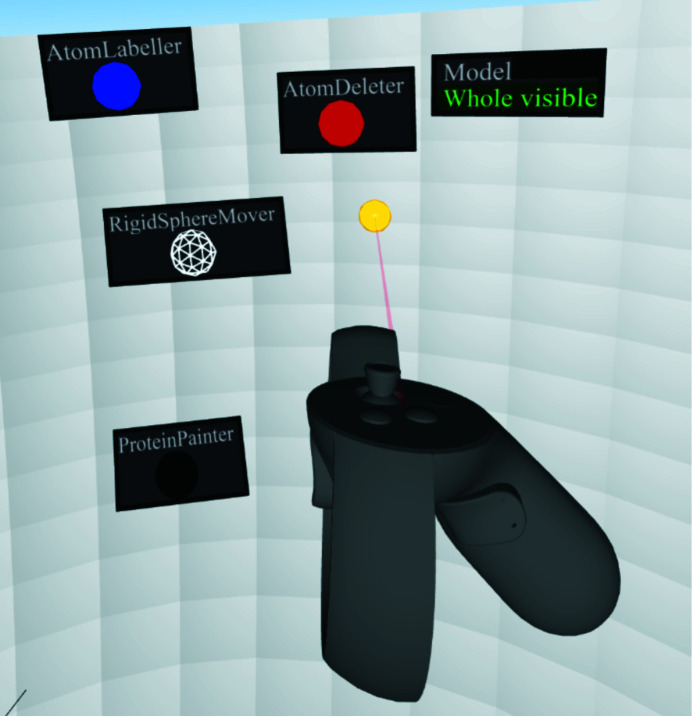
Selection of objects on the panel.

The user has a pair (one per hand) of ‘cursors’ that sit on the panel and can be used to ‘click on’ the objects that sit on the panel (see Fig. 4 ▸). If the user puts the yellow ball over the ‘ProteinPainter’ tool and presses the trigger button, the tool will teleport into their hand until they press the trigger button again. Note that the lasers come out of the side of the hand rather than the front; this worked well because the front is where the model always appears to be, since it is where the clipping slab is.

The user has the ability to move windows on the panel to wherever they would like to put them.

## 3. Map visualization   

The central visualization in model building is one or more molecular models, together with one or more electron-density maps, superimposed. When implementing this in VR (or at least when trying to take advantage of VR in its implementation), there was a need for a divergence from the visualization method in *Coot*.

**Figure 5:**
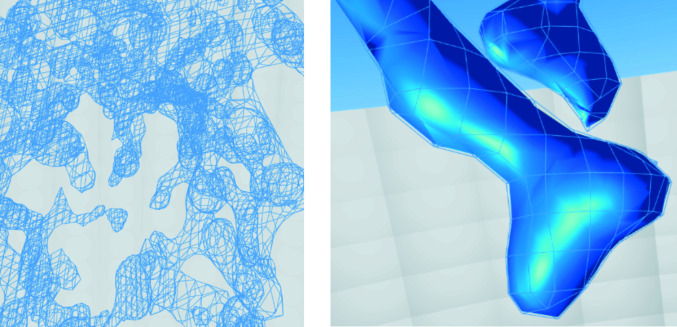
Left: ‘chickenwire’ representation. Right: our method.

Initially, the *Coot* style of surface presentation, the famous ‘chickenwire’ of Fig. 5 ▸, was copied. However, this was un­satisfactory. In *Coot*, it is necessary to have the ‘clipping slab’ (see ‘selective visibility’ below) be quite thin, and only really be looking at a few shapes within density. In VR, it is possible and desirable for it to be thicker, and with a thick chunk of density rendered in the ‘chickenwire’ style, it is very difficult to see what is behind what (see Fig. 5 ▸).

**Figure 6:**
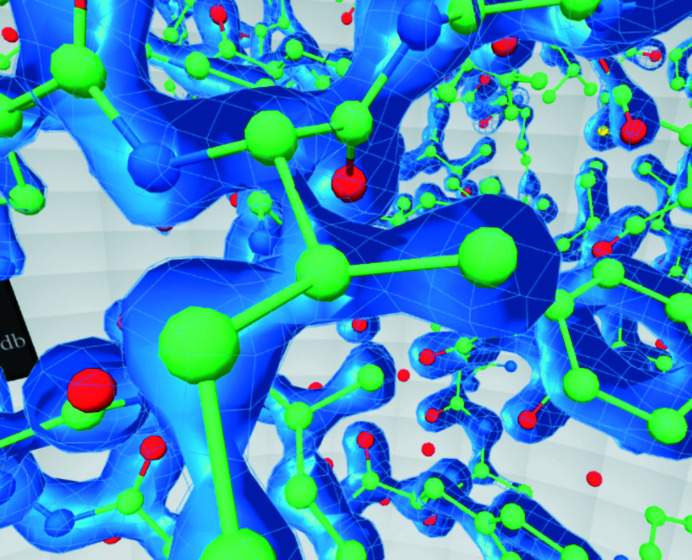
The contour surface around a model in *CootVR*.

Therefore, our representation is a combination of a chickenwire ‘front’ and a solid ‘back’ (Fig. 6 ▸). This representation has attracted positive comments in the structural biology community. It allows the user, at a glance, to see very clearly which atoms are ‘inside’ the surface.

### 3.1. Selective visibility (‘clipping planes’)   

Proteins are intricate three-dimensional shapes, and model building involves inspecting every single part of them very closely, including their interior. The user must be empowered to quickly choose which parts they want to see, because they do not want to see the whole thing at all times.

**Figure 7:**
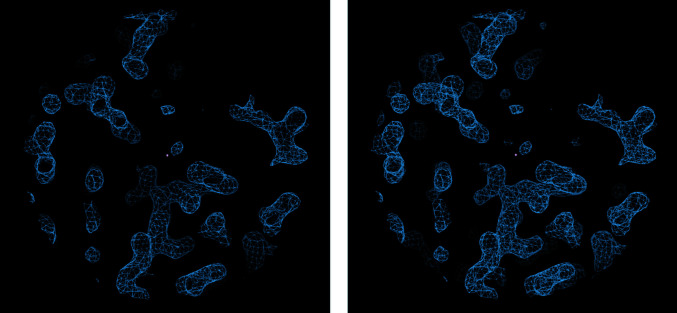
A map displayed in *Coot* with different values of ‘Clipping plane depth’. On the right, the clipping planes are further apart, so one can see more of the map extending backwards and forwards.

In the context of *Coot*, selective visibility is accomplished by controlling the ‘clipping planes’ or the ‘clipping slab’ (see Fig. 7 ▸). Changing the clipping-plane depth is a command bound to the ‘d’ and ‘f’ keys (these keys sit directly beneath the resting position of the left hand, showing how commonly they are used). If a user is working on a 3D object in the real world (a Lego model, for example), it is obviously quite common that a person would want to look at something behind or in front of what they are currently looking at. On a 2D screen this has to happen quite slowly.

**Figure 8:**
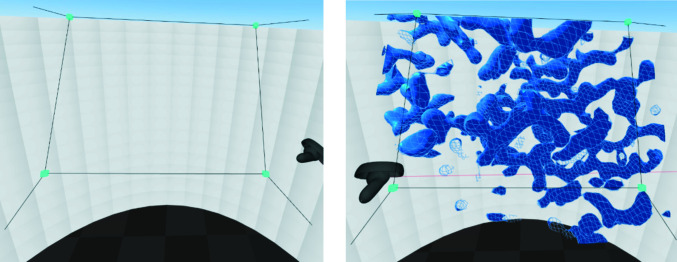
*CootVR*’s clipping volume without (left) and with (right) something inside it.

Our approach is depicted in Fig. 8 ▸. The molecule and map are visible within a specific 3D area that is enclosed by line segments. The area is in the shape of a ‘frustum’: essentially, a square-based pyramid that is cut off at the top. The peak of the pyramid is the place where the user puts their eyes, *i.e.* the part that they look at is the flattened top of the pyramid. In the context of *Coot* and other molecular-graphics programs, this top is called the ‘front clipping plane’.

At the corners are small blue cubes which can be grabbed and moved if the user wants to change the horizontal, vertical or lateral size of the volume. If a blue cube is grabbed and moved left or right, the cube above or below it will move with it. Additionally, the cube that is horizontally across from it will mirror its movement, maintaining a vertical line of symmetry down the middle. If the cube is moved towards, or away from, the place where the user’s face is expected to be, all of the cubes will move in the same way (equivalent to clipping slab thickness change in *Coot*). This automated movement is very useful; a sophisticated change to the volume can be made with a single hand movement.

**Figure 9:**
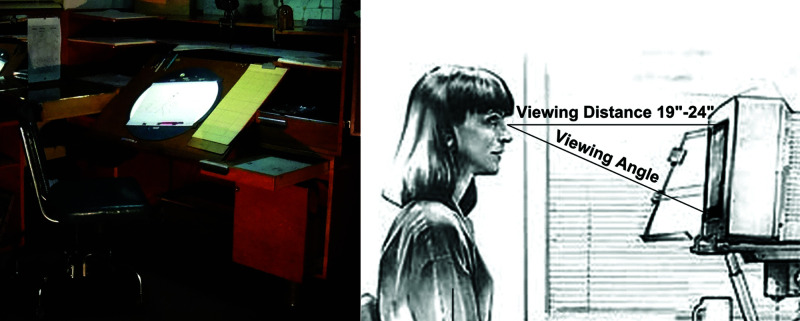
Left: artist’s desks are almost always tilted towards the expected location of their head (Disney artist’s desk, The Conmunity - Pop Culture Geek from Los Angeles, CA, USA, CC BY 2.0, via Wikimedia Commons). Right: ergonomic guidelines emphasize that users’ eyes, in general, are pitched downwards (Yamavu, CC0, via Wikimedia Commons).

With regard to its placement, it is best to have the volume tilted such that the front clipping plane is facing upwards, in accordance with well understood ergonomics (see Fig. 9 ▸).

## 4. Hand tools   

One interesting benefit of the VR platform for model refinement is that the user can interact with the software using both the position and the orientation of their hands. Below, the tools that benefit the most from hand movement are described. All of the tools are ‘big-picture’ movements of large numbers of atoms, *i.e.* at least one residue. This is to be expected; smaller groups of atoms (a single side chain for example) can have their conformation worked out automatically with chemical constraints, while larger groups might have more uncertainty associated with them, *i.e.* there may be more room for the user to be vague about where things should be: hand movements cannot be expected to be precise to within a single angular degree. It is acceptable in the course of model refinement to have a model temporarily be not exactly correct: it is a ‘price worth paying’ in order to be able to quickly examine different possibilities that are ‘vaguely correct’. If these possibilities, which will mostly be wrong, are not at least examined, it is possible that a superior model-fit situation will not be considered, or that it will take longer to find, because a lot of time must first be spent ruling out incorrect possibilities (because they must be considered in great detail).

### 4.1. Examining the model and map with different orientation, position and scale   

The simple act of examining the model and map with one’s hands, in order to see it in different positions and orientations, is an obvious use of VR; having six degrees of freedom, it is fundamentally more capable than the mouse, which has two. In *CootVR*, when the user ‘grabs’ the model and map, they will become ‘stuck’ to the grabbing hand. If the grabbing hand is moved, the model will move precisely such that the grabbing hand stays in the same position and orientation relative to the model.

Additionally, the user can grab the model with both hands and ‘scale’ (with the right hand being the center of the scaling; this allows easy control over one’s ‘focus’). This is somewhat analogous to a ‘zoom’ on an ordinary screen (especially within *Coot*).

The purpose of rotating and moving the model and map in *Coot* and *CootVR* is (obviously) to change what one is seeing, and the user can achieve this goal more quickly in *CootVR*. Even for an expert user of the *Coot* view controls, it takes longer to get to the view that one precisely wants than with *CootVR*. We would estimate that during *Coot* use around 1–2% of the user’s time is spent adjusting the view, although this goes up to 5 or 6% if the ‘*Coot* shake’, which *CootVR* eliminates, is included.

In *CootVR* a user can also ‘multitask’ to a great extent. Using other hand tools, it is very natural and fast to be doing something to the model with one hand, while the other hand is holding it and moving it such that the doing hand is in the easiest place possible and the head has a good view of what the hands are doing. This is in contrast to *Coot*, where view changes are something that must be peformed between tasks: potentially it is the case that what should be a single movement is broken up into many movements by ‘*Coot* shakes’. On this front, therefore, *CootVR* is a considerable speedup.

### 4.2. The ‘rigid mover’ hand tool   

**Figure 10:**
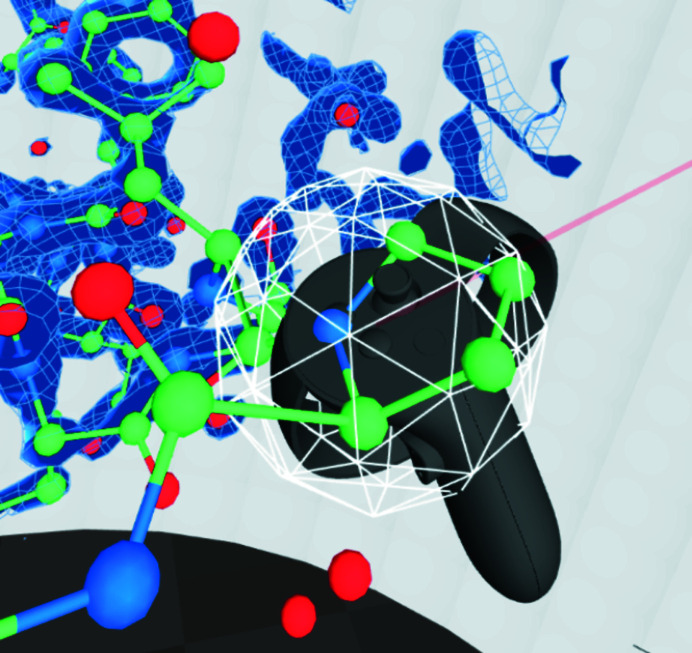
The user’s right hand, holding a histidine side chain.

‘Rigid’ motion is when an object moves as a ‘rigid body’, *i.e.* as if it is completely frozen and no part of it is moving with respect to any other part. It is used sometimes in *Coot* for, for example, moving ligands. Rigid motion has been implemented in *CootVR* using the hand controllers. This allows the user to perform a rigid motion with, in principle, any set of atoms (Fig. 10 ▸).

Rigid motion is an extremely simple kind of movement and is completely insensitive to context. Rigid motion is very likely to cause problems, for example steric clashes or C^β^ deviations. However, these can be dealt with subsequently; in general, the point of rigid movement is to put atoms in a position that is approximately better on the whole and then perform more detailed work to deal with the problems that arise. One example is, again, ligands, where it is extremely common for the user to want to pick up a specific set of connected atoms within a region and rotate and translate them.

To give a more interesting example, one may be considering the fit of a homology structure into a map and have the vague feeling that an α-helix ought to be moved. Doing this ‘properly’, in the sense of carefully making sure that every amino acid fits, may be worthwhile, but it is not yet clear that this would be worth the investment of time. All that is desired, at least while the user is starting out, is to see whether there is room for an α-helix in a given place. The rigid mover allows this hypothesis to be tested in moments, whereas previously it might take at least 5 min.

Two different area-selection methods are available in *CootVR*: sphere selection and chain selection. With sphere selection, when the user presses the grab button they will be holding a set of atoms that are inside a sphere. The sphere is cage-like so that it is obvious which atoms are contained within it. The chain-selection tool works differently: the user must put both of their hands somewhere on the chain, at the start and end of the part of it that they would like to move. All of the amino acids ‘between’ the positions of their hands on the chain become selected, and they can move the chain around as desired.

With the spherical rigid mover tool, it is important for the user to be able to choose the number of atoms that they are grabbing. In order to do this, the model must be scaled down so that the desired set of atoms fit in the sphere.

### 4.3. The ‘protein painter’ tool   

**Figure 11:**
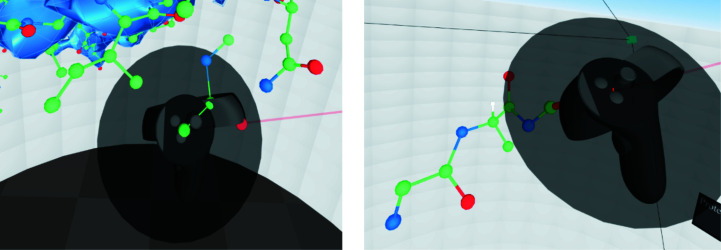
The protein painter about to lay down a first amide (left) and in the process of being used (right).

Since a protein is a chain coiled in a particular way in 3D space, a very obvious way to create them is for a person to move their hand through the air, tracing out its shape (‘painting’ it). This has a clear application within *Coot*: it is relatively common to want to create a chain of a specified length with some specified geometry. One of the first serious tasks identified for *CootVR* was using such a tool (Fig. 11 ▸): it is quite common to have a structure such that there is a bundle of α-helices that are known to be connected in some way but it is not known how. In this situation it may be necessary to try out many possible different ways of connecting up the helices.

**Figure 12:**
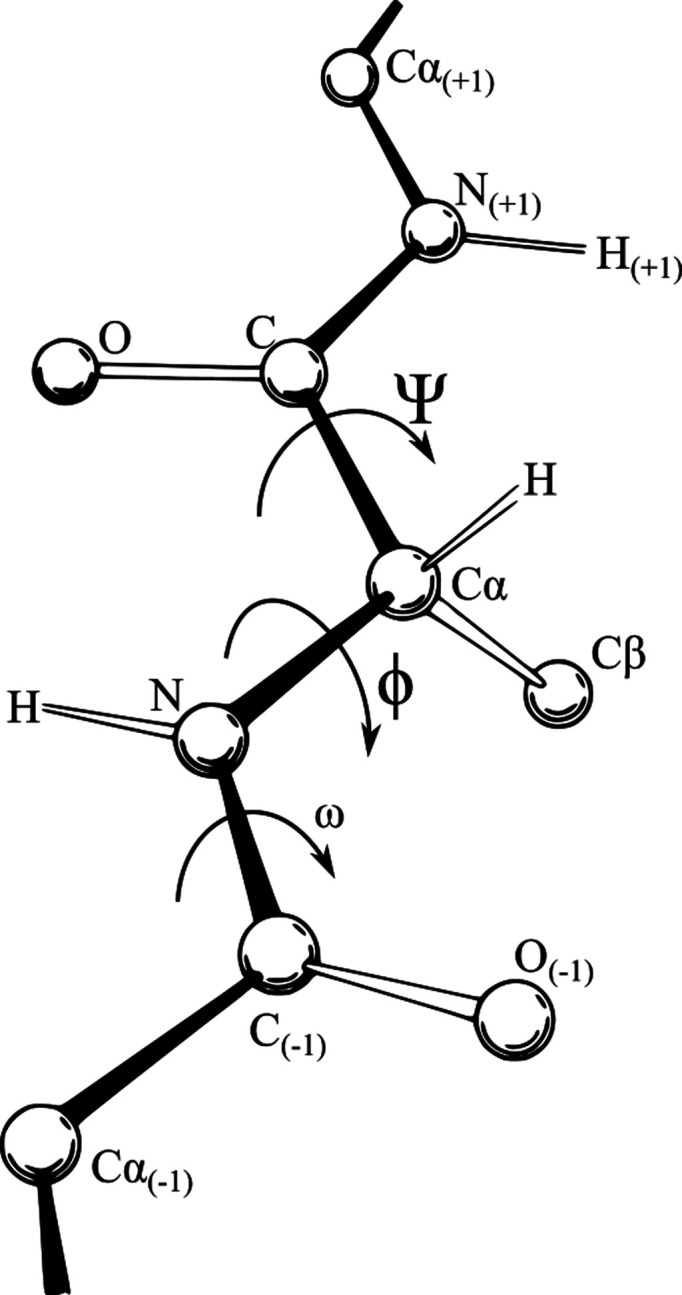
The φ and ψ angles of a backbone (Richardson, vectorized Adam Redzikowski, CC BY 3.0, via Wikimedia Commons).

Amide planes and torsion angles provide a reliable model for considering protein geometry, known as amide planes or ‘torsion angles’; our tool makes use of this formalism (see Fig. 12 ▸ for an illustration). In the formalism, the N—C^α^ bond becomes a natural object to ‘pivot’ around.

The painter tool went through a large number of iterations. It is very easy to use and specific to VR, and has been the main tool in our case studies below. Its design was nontrivial because of the question of exactly how to allow the user to be ‘expressive’ with it, *i.e.* to allow them to create the geometry that they want to create as quickly as possible, but also to enforce the chemical constraints of the φ–ψ formalism.

The way that the painter tool works is as follows.

(i) There is a button that the user may press to create a new amino acid and immediately assume hand control of it. If there is already an amino acid under hand control when the button is pressed, it will become frozen in place, and the new atom will be attached to it with appropriate bond angles.
(ii) Another button reverses the above, essentially being an ‘undo’, deleting the current amino acid and resuming control of the previous one (if it exists).
(iii) If the user moves their hand around, the φ and ψ angles of the currently selected amino acid will change such that its C^α^ atom will be as close as possible to the user’s hand position in 3D space.
(iv) If there are two possible sets of bond angles that will achieve equally close proximity to the hand, there is another button which, if pressed, will switch between these two sets.
(v) While doing this, the user can move their head as they please, and move the molecule too.

#### 4.3.1. Timing test of the protein painter tool   

The general question in this work is ‘how much of an improvement, if any at all, does VR offer over a mouse-and-keyboard interface in the context of structural biology?’. This is too general a question to be answered in one project, but more feasible is the related question ‘is there any common model-building task for which VR is better than a mouse-and-keyboard interface?’. Considering the protein painter tool, this question can be answered in the affirmative.

The experiment was set up by taking unseen data sets with model and map both available and removing some amino acids from the chain. The puzzle was then to fill in the chain in a rough way (which in a real-world situation would then be refined into place with the automatic refinement system in *Coot*), while being timed. This was performed by the authors of this paper, with Paul Emsley standing as an expert *Coot* user and Hamish Todd being the only example of an expert *CootVR* user. The chains were filled in ‘blind’, *i.e.* both authors had not seen the gap prior to the point where they had to start filling it in.

We considered comparing the ‘fit’ with the final data, but decided not to, as the goal of drawing the new chain at this stage was not to focus on details but to give a general shape for the chain and then to refine it in detail using other tools afterwards.

In all three cases, on the *CootVR* side, the task was brief enough that no break was needed; this would require undonning and re-donning the headset, which would have taken up a large amount of time.

It should be noted that *CootVR* was quite a lot faster than *Coot* on average (Table 1 ▸), and thus it was concluded that at least for this usage VR is superior to the mouse/keyboard interface.

## 5. Case study   

In order to obtain a subjective sense of what it is like to use the protein painter, and *CootVR* generally, for a protracted length of time, we took it upon ourselves to build a structure. To obtain an accurate, informative model, many validation features are needed, which *CootVR* lacks, and so obtaining an accurate model was not the goal of this case study; the goal was to obtain insight into what can happen when VR is used for model building, which *CootVR* can offer, even in its basic state.

*CootVR* is not suited to the kinds of small adjustments that are needed in crystallographic data, which are mostly automated, so it was decided to focus on cryo-EM. A 2.3 Å resolution, 4368-residue data set for apoferritin released as part of a competition (EM Validation Challenges: 2019 Model Metrics Challenge; https://challenges.emdataresource.org/?q=model-metrics-challenge-2019), EMD-20027, was used.

**Figure 13:**
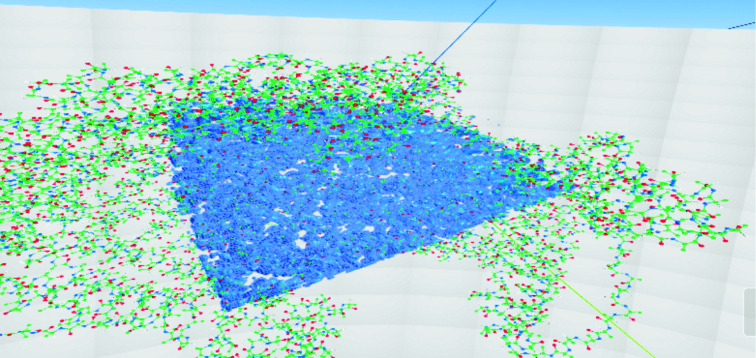
Our case-study protein in a state of partial completion.

The study took around 9 h spread over several days. The entire data set was filled with backbone (Fig. 13 ▸). Although the apoferritin proteins are related by symmetry, this was not used (because the purpose of the exercise was to spend a lot of time using *CootVR*).

A number of interesting observations were made. It had previously been suggested that α-helices (of which apoferritin has many) would be difficult to deal with in the context of *CootVR*, but actually it turned out that they were extremely easy, more easy than nonhelix areas of protein. The reason it was believed that they would be difficult was because α-helices, in the context of non-VR *Coot*, have been reported to be difficult to build by adding amino-acid residues one by one. This is probably owing to the fact that α-helices are innately 3D objects. In the context of *CootVR*, the reason that they are so easy to build is because they are very repetitive; there is one specific motion with the hand to rotate the model as desired and then another to place the amino acid.

Interestingly, with many amino-acid placements, it was barely even necessary to make a second hand movement: one can simply hold one’s ‘painting hand’ in place and rotate the model such that it is in the correct position relative to the rest of the model with a bottle-unscrewing motion. The thing that ends up happening ‘naturally’ is that the molecule-moving hand would take care of large movements while the painting hand takes care of minor movements. This was effortless to get used to.

In using *CootVR*, we found the following.

(i) It is necessary to rest one’s elbows on a hard surface; when one does not do this, one’s wrists and upper arm will rapidly become fatigued.
(ii) It was good to keep interactions to bouts of approximately 15–25 min. Any more could cause eye strain, and more prolonged sessions of 50 min induced neck strain. This may have been owing to dryness inside the headset, or possibly because wearing a VR headset can cause a reduction in the amount that people blink.
(iii) It was good to keep the model very close to the face, within around 25 cm, probably because (unconsciously) it is useful to obtain as much information as possible from stereoscopy (this may have contributed to the aforementioned eye strain). It is interesting to note that if this is a requirement for VR-based model building then light-field displays may not be a very good replacement for headsets, and that solving the vergence-accomodation problem could be beneficial for structural biology (Vision, Oculus Developer Guidelines; https://developer.oculus.com/learn/bp-vision/).
(iv) As with other VR programs, social interaction, drinking coffee or tea, using other pieces of software and reading papers were hard to combine with using *CootVR* owing to the problem of needing to don and un-don the headset, which could take 20–60 s at a time.

## 6. Conclusion   

*CootVR* allows biomolecular models to be built in virtual reality. It is not a practical proposition for this use currently, but offers some tool designs that may be useful if implemented at a later stage in the development of VR.

There was one opportunity for interesting design exploration that was not pursued, which could be be picked up by another group, which is putting many many more validation visualizations on the molecule: VR permits this, as more visual information can be taken in. The *Coot* ‘environment distances’ tool has been implemented in *CootVR*, but as in *Coot* it can only be applied to a few residues at a time: this makes sense on a conventional display, because otherwise distracting visual clutter will be created. However, with VR large amounts of extra detail can be added without being visually distracting, meaning that environment distances could theoretically be turned on everywhere. This can save time, because it removes decision making about where the environment distances should go. Model building is not the only thing for which *Coot* is used: it is also useful for ‘analysis’, *i.e.* examining the model and figuring out what information it gives about the activity of the protein. *CootVR* may in principle be better for analysis than *Coot* because it involves a great deal of view adjustment and examination, although no moving of atoms and therefore not much hand usage apart from the view adjustment.

Finally, we would cautiously claim that these results have some implications beyond structural biology. Within scientific visualization there is a large amount of complex 3D data that it is worthwhile to visualize, for example anatomical MRI scans and tensor fields. The selective visibility system implemented in *CootVR* may also be applicable to these domains.

## Supplementary Material

Video demonstrating features of CootVR. DOI: 10.1107/S2059798320013625/ir5009sup1.mp4

## Figures and Tables

**Table 1:** *CootVR* compared with *Coot* for *ab initio* chain construction

Puzzle	No. of residues	*Coot* result (s)	*CootVR* result (s)
1	4	36	30
2	8	180	25
3	25	1140	72
